# Protective effects of Chinese Fenggang zinc selenium tea on metabolic syndrome in high-sucrose-high-fat diet-induced obese rats

**DOI:** 10.1038/s41598-018-21913-w

**Published:** 2018-02-23

**Authors:** Jie Yu, Jing Yang, Mizhuan Li, Xuesong Yang, Pan Wang, Jie Xu

**Affiliations:** 1 School of Public Health, Zunyi Medical University, Zunyi Guizhou, 563099 P.R. China; 2 Department of Nuclear Medicine, The Affiliated Hospital of Zunyi Medical University, Zunyi Guizhou, 563099 P.R. China

## Abstract

The protective effect of zinc selenium tea against metabolic syndrome (MetS) was tested by using a high-sucrose-high-fat diet (HSHFD)-induced MetS model. Fifty Sprague–Dawley rats were randomly divided into five groups: normal diet (C-group), HSHFD (CH-group), HSHFD + green tea (0.24 g/kg/day) (TH-group), HSHFD + low-dose zinc selenium organic tea (0.24 g/kg/day) (ZTHL-group), and HSHFD + high-dose zinc selenium organic tea (1.20 g/kg/day) (ZTHH-group). After 8 weeks, compared to both the C-group and CH-group, the hepatosomatic index (HI) was significantly reduced in the ZTHL-group (p < 0.05). Fasting blood glucose (FBG) levels were highest in the TH-group, followed by the CH-group, then the ZTHL-group, then the ZTHH-group, and finally the C-group. Compared with the CH-group, the serum total cholesterol (TC) and low density lipid-cholesterol (LDL-C) concentrations were significantly lower in the ZTHH-group (p < 0.05). Significant decreases in serum alanine aminotransferase (ALT), aspartate aminotransferase (AST), total bile acids (TBA), alkaline phosphatase (ALP), and direct bilirubin (DBIL) levels were observed in ZTHL-group versus the CH-group (p < 0.05). Serum alpha-L-fucosidase (AFU) levels in the ZTHH-group were lower than in the CH-group (P < 0.01). Histopathological examination of the liver and fat biopsies illustrates that the liver cells showed a decrease in the extent of necrosis and dropsy in the ZTHL-group and ZTHH-group versus the CH-group. Zinc selenium tea showed a protection effect against hepatic damage.

## Introduction

Tea, from a biological standpoint, is a mixture of a large number of bioactive compounds including catechins, flavonols, lignans, and phenolic acids. Studies have also indicated that tea and its constituents may have properties that help to prevent cancer, metabolic syndrome (MetS), and other disease states^1,2^. The three main types of tea are green, oolong, and black, which differ in terms of their processing and chemical composition. Green tea is the most widely consumed beverage worldwide, following water^3^. Increasing evidence suggests that green tea and its bioactive polyphenolic compounds can significantly ameliorate certain features of metabolic syndrome and help to reduce the subsequent risk of developing type 2 diabetes mellitus and cardiovascular disease (CVD)^4,5^. Fenggang zinc selenium tea is a type of green tea. Fenggang zinc selenium tea originated in Fenggang county, Zunyi city, in the Guizhou province southwest of China. The tea is well known for being rich in organic zinc and selenium, in addition to containing normal levels of green tea polyphenols^6^. Fenggang zinc selenium tea has various health-promoting activities. Several studies have shown that zinc and selenium-rich green teas have positive effects in regards to their antioxidant ability in animals. Zinc and selenium-rich green teas have antioxidant and anti-aging effects, which are comparably stronger than traditional green teas^7,8^.

Metabolic syndrome affects a significant proportion of the population^9,10^ and is becoming increasingly more prevalent in developing countries, such as China where the prevalence is 9.8% in women and 17.8% in men^11^. The syndrome embodies many components, including central obesity, insulin resistance, dyslipidemia, and hypertension^12^. Contributing factors that predispose someone to MetS can be hereditary or environmental and include the following: a family history of diabetes, specific ethnic backgrounds, senescence, sedentary lifestyles, and unhealthy eating habits^2,9,10,13^. Of these, one of the primary causes of the increasing prevalence of MetS is the excessive consumption of high-fat and high-sucrose foods^14^.

The metal zinc is the second most common trace metal in the body and, as an essential micronutrient, plays many important roles in biological systems. Zinc also plays crucial roles in the synthesis, storage, and secretion of insulin and in the actions of insulin on carbohydrate metabolism^15^. Zinc affects lipogenesis more noticeably in chemically-induced diabetes than in normal tissue. Zinc can induce an increase in glucose transport into cells and potentiate insulin-induced glucose transport, likely acting through the insulin-signaling pathway^16^. Prior epidemiological studies in China and Tunisia reported that plasma zinc level in type 2 diabetic (T2DM) subjects was significantly decreased, which means as the zinc centration decreasing it was more likely to develop type 2 diabetes^17,18^. Previous research studies have been conducted to clarify the molecular mechanisms underlying the action of Zinc in MetS^15,16^.

Selenium (Se) is an essential trace element, which is important for human health, as it is involved in various physiological and ecological processes, such as hormone and insulin metabolism. Elevated levels of serum selenium have been found to be associated with MetS^19,20^. Epidemiological studies have shown that metabolic syndrome is associated with higher plasma selenium concentrations in women (odds ratio (OR) Z 1.55(1.28e1.89))^21^. Higher levels of plasma selenium might increase the risk of metabolic syndrome and elevated fasting plasma glucose levels^22^. Furthermore, selenium intake reduces serum C3, an early marker of metabolic syndrome manifestations, in healthy young adults^23^. Wu (2012) demonstrated that high level of serum Zn was relative risk factor for abnormal blood lipids or spectrometry (ABL), and reducing level of serum Zn might have effects to prevent and treat ABL^24^. Therefore, we surmised that maintaining normal serum zinc and selenium level is beneficial for preventing MetS.

Examining the association between Fenggang zinc selenium tea consumption and metabolic syndrome is necessary to determine if it bestows a beneficial influence on glucose and lipid metabolic disorder and liver damage. Unfortunately, there has been limited research examining whether Fenggang zinc selenium tea consumption affects glucose and lipid metabolic disorder or whether it can improve hepatic function. In this study, the potential beneficial effects of Chinese Fenggang zinc selenium tea on endocrine metabolism in high-sucrose-high-fat diet-induced obese rats were examined.

## Materials and Methods

### Ethics Statement

All procedures were conducted at Zunyi Medical University and were performed in strict accordance with the guidelines and regulations set forth by the Zunyi Medical University ethics committee with full approval from its Animal Care and Use Committee. All experimental protocols were approved by the animal research committee of Zunyi Medical University.

### Animals and Treatments

**Figure 1:**
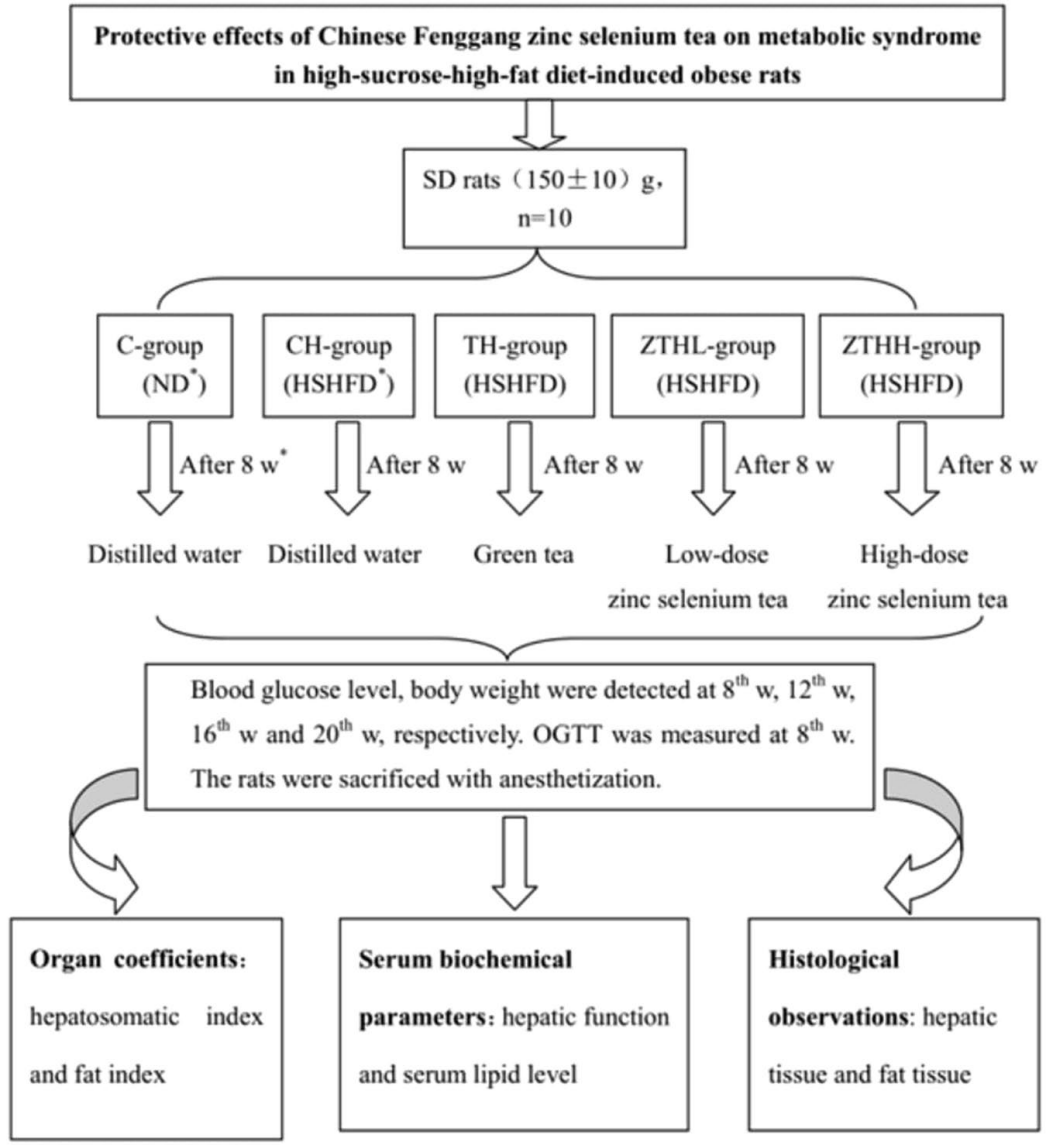
Exposure flow chart. (ND: normal-diet, HSHF: high-sucrose-high-fat diet, W: week).

Fifty Sprague-Dawley (SD) rats purchased from the Animal Center of the Third Military Medical University (Chongqing, China), each with an initial weight of 150 ± 10 g. Following a 1-week acclimation period, the 50 rats, half of which were male, were randomly assigned to 5 groups, each with 10 rats. After the rats had been fed a normal or high-sucrose-high-fat diet (HSHFD) for 8 weeks, they received the test chemical via gavage. The first group, fed a normal diet, received gavage with distilled water and served as the normal-diet control (C-group). The second group fed an HSHF diet received gavage with distilled water (CH-group). The third group fed an HSHF diet received gavage with green tea (0.24 g/kg/day) (TH-group). The fourth group fed an HSHF diet received gavage with low-dose zinc selenium organic tea (0.24 g/kg/day) (ZTHL-group). The fifth group fed an HSHF diet received gavage with high-dose zinc selenium organic tea (1.20 g/kg/day) (ZTHH-group). One kilogram of zinc selenium organic tea dry matter contains 40–100 mg zinc and 0.25–3.50 mg selenium^25^. The rats were fed the experimental diets for 8 weeks. Body weights were monitored every week. Fasting blood glucose (FBG) was monitored every 4 weeks. The animals were maintained under controlled temperature (20 ± 1 °C) and humidity (60 ± 5%), on a 12-hr light (09:00–21:00 hr), 12-hr dark (21:00–09:00 hr) cycle. Food and water were freely available. All procedures involving the use of laboratory animals were performed in accordance with the Animal Care and Use Guidelines in China. The flowchart depicting exposure protocols and time duration of exposure is shown in Fig. 1.

Animals received treatment once daily by gavage, 6 times a week, continuously for 12 weeks. Blood samples were taken at 13 weeks from 12 - h fasted rats. These blood samples were used for serum glucose and lipid measurements. Serum total cholesterol (TC), triglyceride (TG), high-density lipoprotein cholesterol (HDL-C), and low-density lipoprotein cholesterol (LDL-C) concentrations were determined in the endpoint plasma samples. At the end of the study, the rats were sacrificed with intraperitoneal chloral hydrate (100 g/L) following a 12-h fast. Pancreas, liver, and adipose tissues (perigonadal fat pad) were removed, rinsed with phosphate-buffered saline, and weighed. These samples were stored at −70 °C.

### Preparation of zinc selenium tea decoction or infusion

The zinc selenium tea aqueous extract was prepared according to the method presented by Snoussi^3^ from Chinese Fenggang zinc selenium tea (organozinc content is 40–100 mg/kg, and organoselenium is 0.25–3.50 mg/kg), which was supplied by Wanhuyuan zinc selenium tea Ltd., Fenggang county, Guizhou province, China. Ordinary green tea was supplied by Weidao tea Ltd., Guizhou province, China. The tea leaves (100 g) were ground and then boiled in double-distilled water (1000 mL), followed by stirring for 15 minutes at 70 °C; the procedure was repeated 3 times. Collected extracts were centrifuged after filtration (2700 g^−1^, 15 minutes) and then lyophilized under vacuum (Multi Branch Trade & Manufacturing Company “Elena,” Zelazkow, Poland).

### Hepatosomatic index (HI) and fat index

The hepatosomatic index (HI) and fat index to body weight ratios were calculated according to the formula: organ weigh/body weight × 100%. HI = liver weight/body weight × 100%. Fat index = the weight of epididymal fat/body weight × 100%.

### Oral glucose tolerance test (OGTT experiment)

Oral glucose tolerance tests (OGTT experiment) were performed at 8 weeks. The rats were fasted overnight (16 h, 6:00 pm to 10:00 am) prior to a single oral administration of 1 mL of 2 g/kg of glucose. At each time point (0, 30, 60, and 120 min) after administration, an aliquot of blood was taken from the tail vein, being immediately subjected to blood glucose level (BGL) measurement with a disposable glucose sensor (Glutest Pro, Sanwa Chemical Research Co., Tokyo, Japan). Plasma was separated from the remaining blood samples by centrifugation (4 °C, 3500 × g, 15 min) and stocked at −30 °C until further use^4^.

### Serum biochemistry

Fasting blood glucose levels were assayed by collecting a drop of blood from the tail vein using a glucometer and paper. Total cholesterol (TC), triglyceride (TG), high density lipoprotein cholesterol (HDL-C), and low density lipoprotein cholesterol (LDL-C) serum concentrations were determined in a Cobas 8000 modular chemistry analyzer (Roche Co., Mannheim, Germany). Serum alanine aminotransferase (ALT), aspartate aminotransferase (AST), γ-glutamyl transpeptidase (GGT), alkaline phosphatase (ALP), total bilirubin (TBIL), direct bilirubin (DBIL), indirect bilirubin (IBIL), total bile acid (TBA), Cholinesterase (CHE), and alpha-L-fucosidase (AFU) were assayed using a commercially available clinical test kit with a biochemistry analyzer system (Synchron Clinical System Lx 20; Beckman Coulter Inc, Fullerton, CA, USA).

### Histological observations of hepatic tissue and fat tissue

Sections of liver and adipocyte samples were fixed in a buffer solution containing 10% formalin and processed for paraffin embedding. Briefly, 4 - μm sections were stained with hematoxylin-eosin and observed under light microscopy (Olympus BX41) with the magnifying power of 400×^5^.

### Statistical analysis

The statistical analyses were performed with SPSS software, version 13.0 for Windows (SPSS Inc., Chicago, IL). Values of all variables are presented as mean ± SEM. One-way analysis of variance (ANOVA) with Tukey’s HSD post-hoc test and LSD-t test were used to determine the effects of different treatments. A P-value < 0.05 was considered statistically significant.

## Results

### Effect of zinc selenium tea on body weight, HI, and fat index in rats in experimental groups

#### Body weight

**Figure 2:**
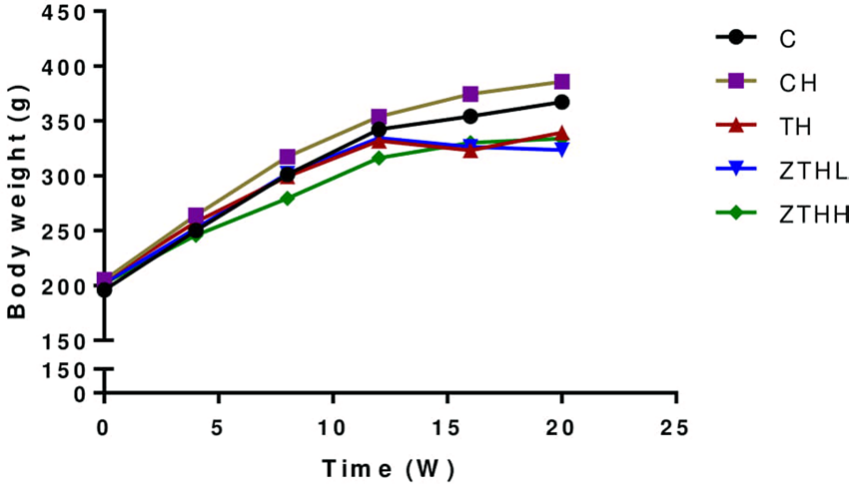
Effect of zinc selenium tea on the body weight in experimental groups (g, mean and SEM, n = 10).

Figure 1 shows the changes in body weight for each experimental group. Before 8 weeks, the body weight increased markedly in the CH-group versus the C-group, but this difference was not significant. After 8 weeks, the body weight increased slightly in the TH-group, ZTHL-group, and ZTHH-group versus the CH-group. The TH-group, ZTHL-group, and ZTHH-group had lower mean body weights than the CH-group, but no significant difference was found among the four groups (*p* > 0.05, Fig. 2).

#### Hepatosomatic index

**Figure 3:**
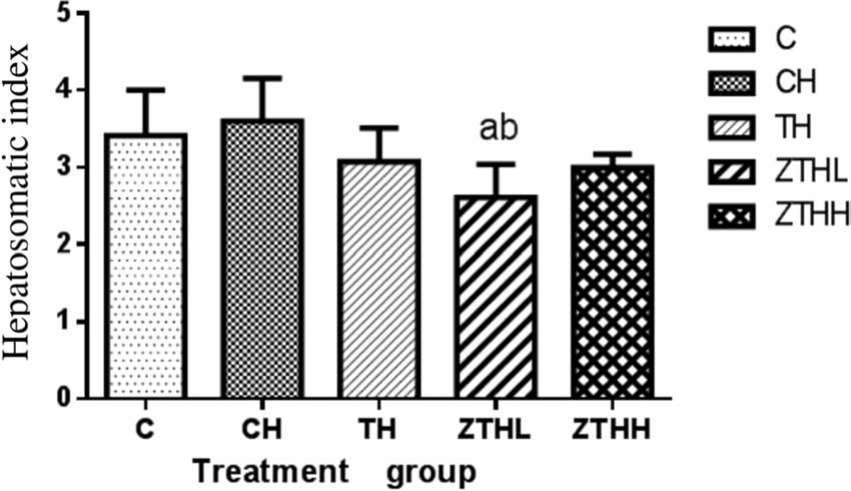
Effect of zinc selenium tea on hepatosomatic index (%, n = 10, mean and SEM) in experimental groups. ^a^Vs C-group, *p* < 0.05; ^b^vs CH-group, *p* < 0.05.

At 20 weeks, in comparison with both the C-group and CH-group, the HI was significantly reduced in ZTHL-group (*p* < 0.05). The HI was increased in the CH-group versus the C-group, but this difference was not statistically significant (*p* < 0.05, Fig. 3).

#### Fat index

**Figure 4:**
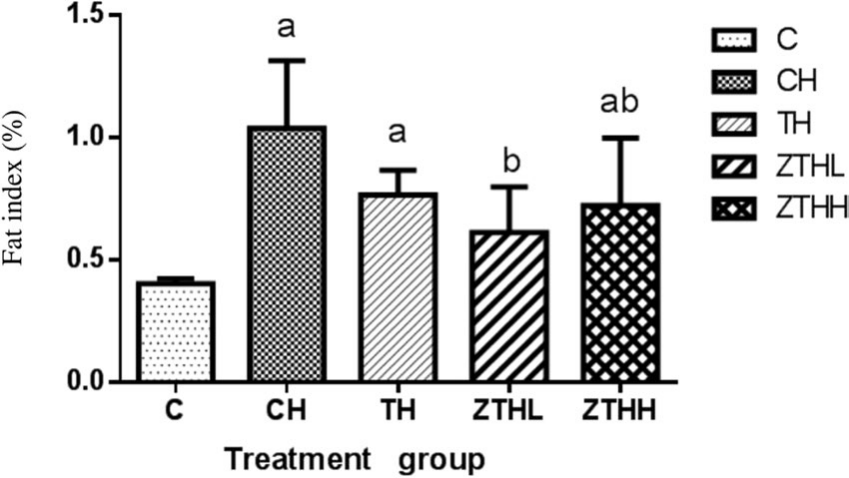
Effect of zinc selenium tea on fat index (%, n = 10, mean and SEM) in experimental groups. ^a^Vs C-group, *p* < 0.05; ^b^vs CH-group, *p* < 0.05.

At 20 weeks, the fat index was significantly increased in the CH-group, TH-group, and ZTHH-group compared to the C-group (*p* < 0.05). The fat index was significantly decreased in both the ZTHL and ZTHH-group versus the CH-group (*p* < 0.05). In addition, the fat index was decreased in the ZTHL-group compared to the ZTHH-group, but this difference was not statistically significant (*p* > 0.05, Fig. 4).

### Effect of zinc selenium tea on glucose metabolism in different rodent experimental groups

#### Fasting blood glucose (FBG)

Before 8 weeks, the CH-group, TH-group, and ZTHL-group had significantly higher FBG levels than the C-group (*p* < 0.05). At 16 weeks, the TH-group had significantly higher FBG levels compared to the C-group (*p* < 0.05). Both the ZTHL-group and ZTHH-group had significantly lower FBG levels compared to the TH-group (*p* < 0.05). At both 16 and 20 weeks, the TH-group had significantly higher FBG levels than the CH-group (*p* < 0.05). At 8, 16, and 20 weeks, the FBG levels exhibited the following trend: TH-group > CH-group > ZTHL-group > ZTHH-group > C-group (Table 1).

**Table 1:** Effects of zinc selenium tea on the FBG level in experimental groups (mean and SEM) (mmol/L).

Group	Case (n)	0 W	8 W	16 W	20 W
C	10	4.70 ± 0.07	4.44 ± 0.22	4.56 ± 0.22	4.08 ± 0.29
CH	10	4.98 ± 0.04^a^	4.68 ± 0.22	4.85 ± 0.18	4.88 ± 0.14
TH	10	5.04 ± 0.06^a^	5.50 ± 1.37^b^	5.53 ± 0.17^a^	5.50 ± 0.20^b^
ZTHL	10	4.99 ± 0.05^a^	4.20 ± 0.22	4.51 ± 0.14^c^	5.00 ± 0.08
ZTHH	10	4.85 ± 0.05	4.55 ± 0.39	4.54 ± 0.23^c^	4.53 ± 0.18

^a^Vs C-group, *p* < 0.05; ^b^vs CH-group, *p* < 0.05; ^C^vs TH-group, *p* < 0.05.

#### Oral glucose tolerance tests (OGTTs)

**Figure 5:**
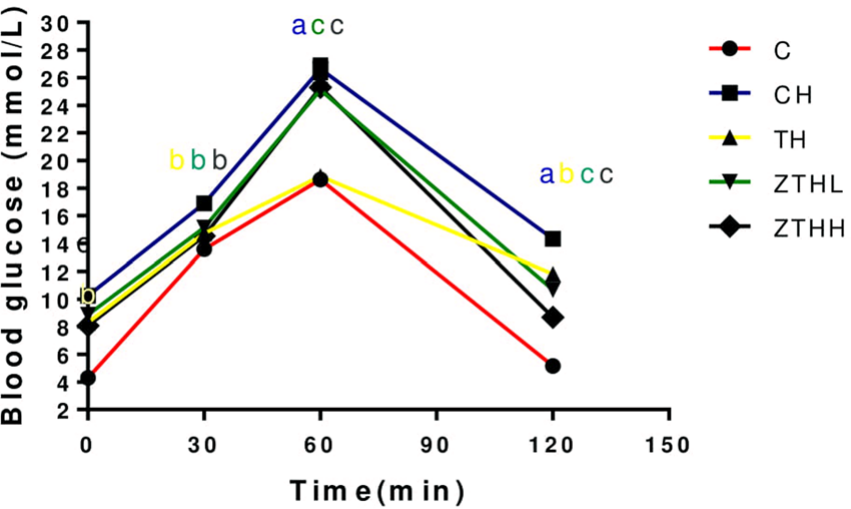
Effect of zinc selenium tea on OGTT in experimental groups (mean and SEM). SEM bars are small and contained within the symbol in the figure.

At 12 weeks, the CH-group, the TH-group, the ZTHL-group, and the ZTHH-group had significantly higher FBG levels compared to the C-group (*p* < 0.05). After OGTT, the two-hour blood glucose level returned to normal in the C-group. The TH-group, ZTHL-group, and ZTHH-group had higher two-hour blood glucose levels compared to their FBG levels (*p* < 0.05, Fig. 5).

**Figure 6:**
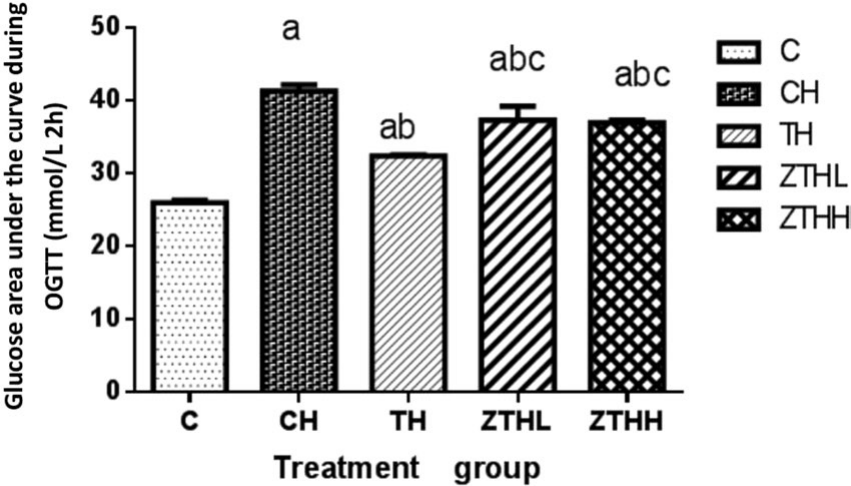
Glucose area under the curve during oral glucose tolerance test (OGTT) (n = 10, mean and SEM). ^a^Vs C-group, *p* < 0.05; ^b^vs CH-group, *p* < 0.05; ^c^vs TH-group.

At 12 week, there was significant difference in the area under curve among the treatment groups (F = 128.279, *p* < 0.001). Compared with the C-group, the area under curve was significantly increased in the CH-group (*p* < 0.001), TH-group (*p* < 0.001), ZTHL-group (*p* < 0.001) and ZTHH-group (*p* < 0.001). On the contrary, the area under curve was significantly decreased in the TH-group (*p* < 0.001), ZTHL-group (*p* < 0.001) and ZTHH-group (*p* < 0.001) in comparison to CH-group. The area under curve was significantly increased in the ZTHL-group (*p* < 0.001) and ZTHH-group (*p* < 0.001) when compared to the TH-group (Fig. 6).

### Effect of zinc selenium tea on biochemical measurements in different rodent experimental groups

**Figure 7:**
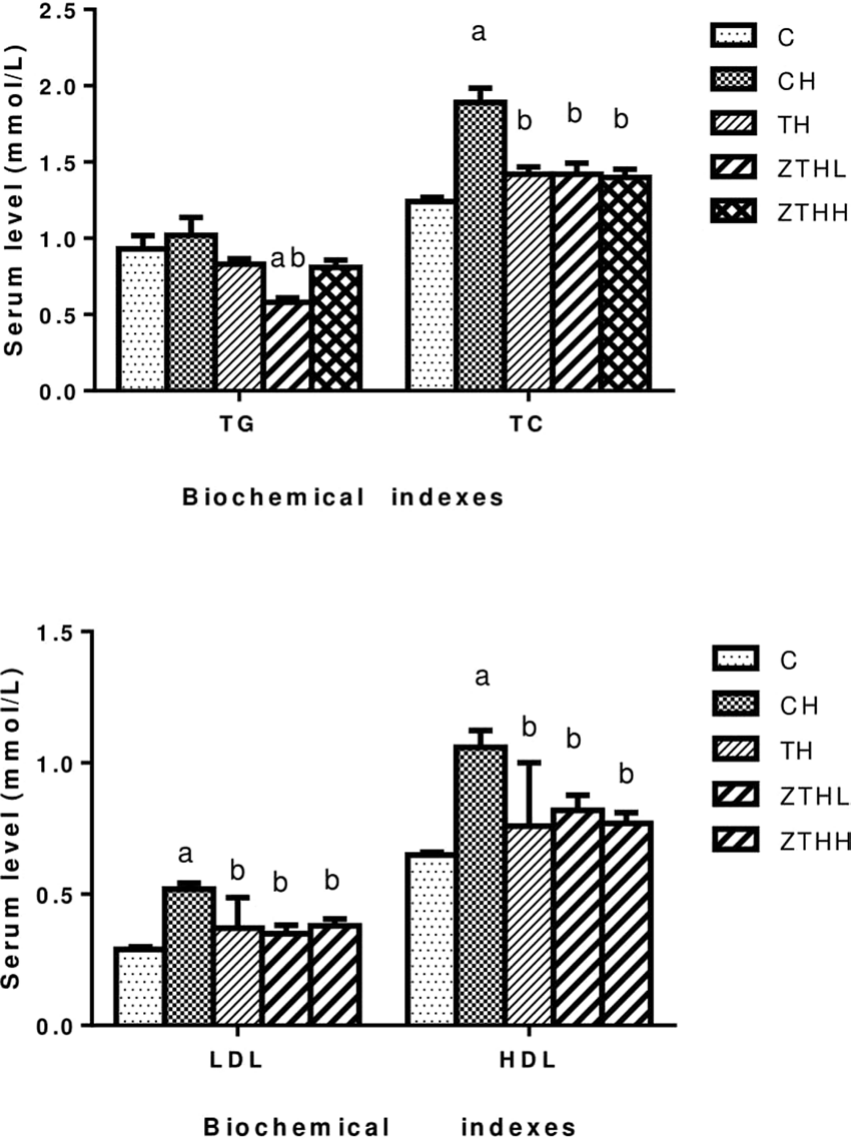
Effect of zinc selenium tea on serum TC, TG, HDL-C, and LDL-C concentrations (n = 10, mean and SEM) in experimental groups. ^a^Vs C-group, *p* < 0.05; ^b^vs CH-group, *p* < 0.05.

Compared with the C-group, the serum TC, HDL-C, and LDL-C concentrations were significantly increased in the CH-group (*p* < 0.05), while the serum TG concentrations in the ZHHL-group were significantly decreased (*p* < 0.05). Compared with the CH-group, the serum TC concentration was significantly lower in the TH-group, ZTHL-group, and ZTHH-group (*p* < 0.05), while the serum TG concentration was significantly lower in the ZTHL-group (*p* < 0.05), and the HDL-C and LDL-C concentrations were significantly lower in the TH-group, ZTHL-group, and ZTHH-group (*p* < 0.05, Fig. 7).

### Effect of zinc selenium tea on serum indices of liver function in different rodent experimental groups

**Figure 8:**
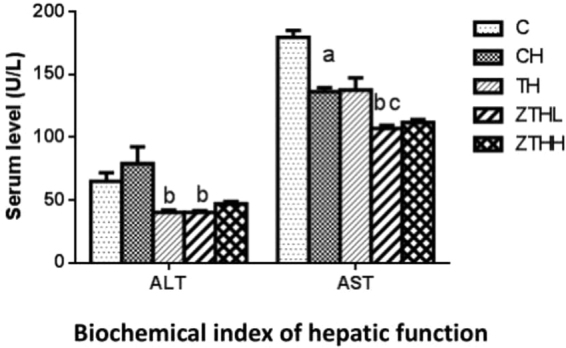
Effect of zinc selenium tea on ALT and AST activities in serum (n = 10, mean and SEM) in experimental groups. ^a^Vs C-group, *p* < 0.05; ^b^vs CH-group, *p* < 0.05.

At 20 weeks, a significant reduction in serum AST levels was observed in the CH-group versus the C-group (*p* < 0.05). A significant decrease in serum ALT levels was observed in both the TH-group and ZTHL-group versus the CH-group (*p* < 0.05). Serum AST levels were lower in the ZTHL-group versus the CH-group and TH-group (*p* < 0.05, Fig. 8).

At 13 weeks, the serum levels of TBIL, DBIL, and IBIL in the ZTHH-group were higher versus the C-group (*p* > 0.05), while the serum levels of DBIL in the ZTHL-group were significantly lower compared to the CH-group (*p* < 0.05, Table 2).

**Table 2:** Effect of zinc and selenium tea on the serum levels of TBIL, DBIL, and IBIL in experimental groups (mean and SEM) (μmol/L).

Group	Case (n)	TBIL	DBIL	IBIL
C	10	2.30 ± 0.13	0.60 ± 0.07	1.70 ± 0.18
CH	10	2.43 ± 0.13	0.80 ± 0.10	1.63 ± 0.07
TH	10	1.68 ± 0.26	0.60 ± 0.04	1.08 ± 0.22
ZTHL	10	1.95 ± 0.23	0.40 ± 0.04^a^	1.55 ± 0.22
ZTHH	9	2.75 ± 0.12	0.70 ± 0.07	2.05 ± 0.15

^a^Vs CH-group, *p* < 0.05.

At 13 weeks, a notable depression in serum levels of TBA was found in both the ZTHL-group and ZTHH-group compared to the CH-group (*p* < 0.05). Similarly, the serum level of ALP in the ZTHL-group was lower compared to the C-group, CH-group, and ZTHH-group (*p* < 0.05, Table 3).

**Table 3:** Effect of zinc and selenium tea on the serum levels of TBA and ALP in experimental groups (mean and SEM).

Group	Case (n)	TBA (μmol/L)	ALP (U/L)
C	10	21.87 ± 2.2	192.20 ± 17.23
CH	10	21.07 ± 2.76	180.00 ± 25.64
TH	10	13.03 ± 2.66	169.20 ± 32.57
ZTHL	10	7.54 ± 0.86^b1^	102.20 ± 13.43^a,b2,c^
ZTHH	9	10.75 ± 0.54^b2^	173.40 ± 17.55^c^

^a^Vs C-group, *p* < 0.01; ^b^vs CH-group, ^b1^*p* < 0.01, ^b2^*p* < 0.05; ^c^vs TH-group, *p* < 0.05.

After 13 weeks, there is no significant difference in serum CHE levels among the C-group, CH-group, TH-group, ZTHL-group, and ZTHH-group. However, serum AFU levels in the CH-group were higher compared to the C-group (*p* < 0.01). Serum AFU levels in the TH-group, ZTHL-group, and ZTHH-group were lower versus the CH-group (*p* < 0.01, Table 4).

**Table 4:** Effects of zinc and selenium tea on the serum levels of CHE and AFU in rats (mean and SEM).

Group	Case (n)	CHE (kU/L)	AFu (U/L)
C	10	0.58 ± 0.13	9.30 ± 0.72
CH	10	0.42 ± 0.05	14.68 ± 0.76^a^
TH	10	0.52 ± 0.13	7.27 ± 0.73^b^
ZTHL	10	0.42 ± 0.76	9.00 ± 0.24^b^
ZTHH	9	0.43 ± 0.07	9.58 ± 0.77^b^

^a^Vs C-group, *p* < 0.01; ^b^vs CH-group, *p* < 0.01.

### Histological examination

#### Histological assessment of liver damage

**Figure 9:**
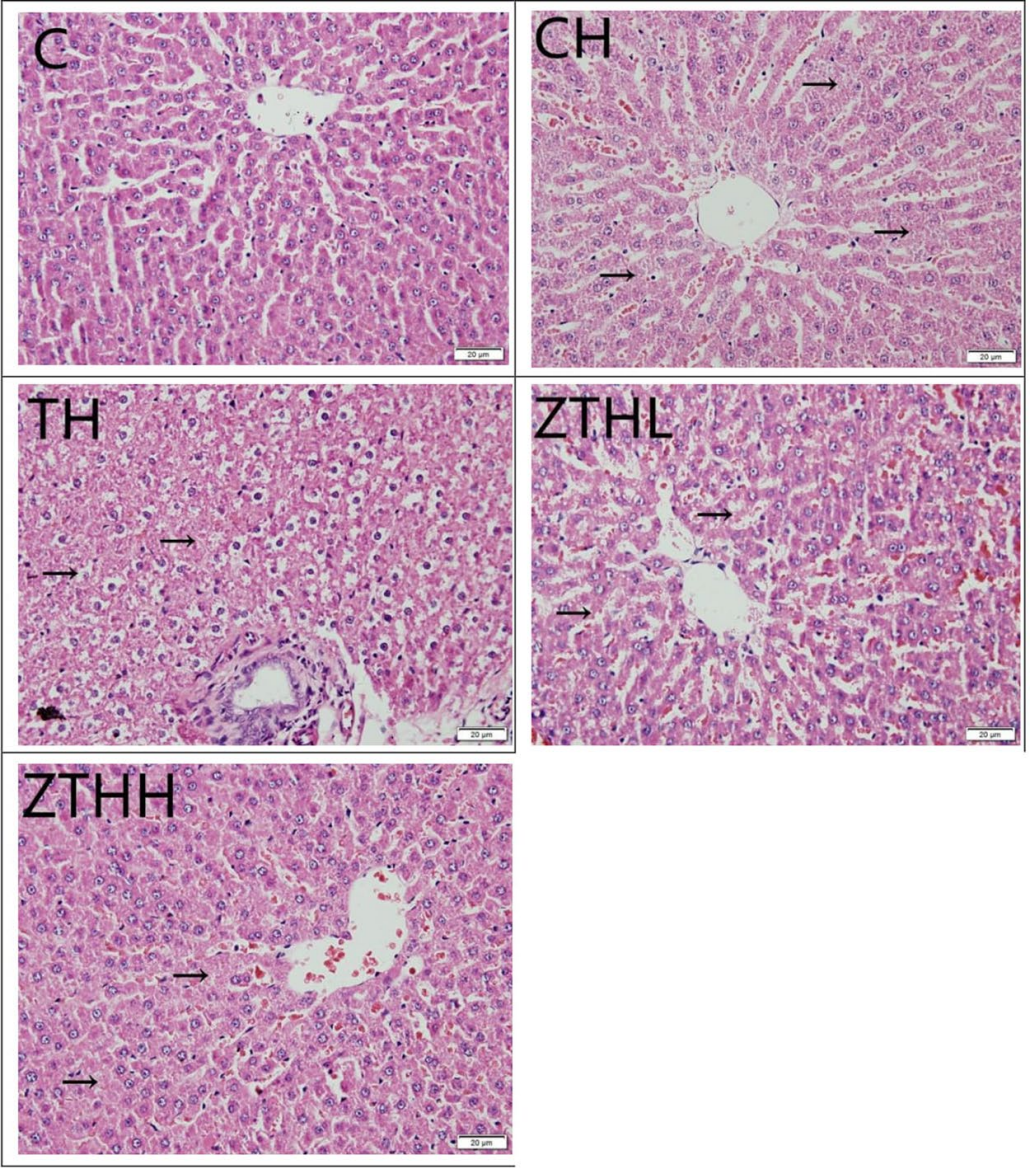
The histological features of liver in experimental rats (400×). Liver tissues from SD rats were stained with hematoxylin and eosin. Arrow: dropsy, necrosis, and disorder of liver cells.

In the C-group, the morphological examination of the tissue stained by H&E showed that the structure of rat liver was clear. The liver cells aligned regularly with central veins, and the center showed a radial pattern. In the CH-group, TH-group, ZTHL-group, and ZTHH-group, there was dropsy and necrosis of the liver cells. The liver cells were disordered. In the TH-group, ZTHL-group, and ZTHH-group, the liver structure recovered to some extent. The liver cells showed a decrease in the extent of necrosis and dropsy as compared to the CH-group. In both the ZTHL-group and ZTHH-group, the extent of necrosis and dropsy decreased dramatically compared to the TH-group, but morphological differences were not obvious between ZTHL-group and ZTHH-group (Fig. 9).

#### Histological assessment of fat tissue

**Figure 10:**
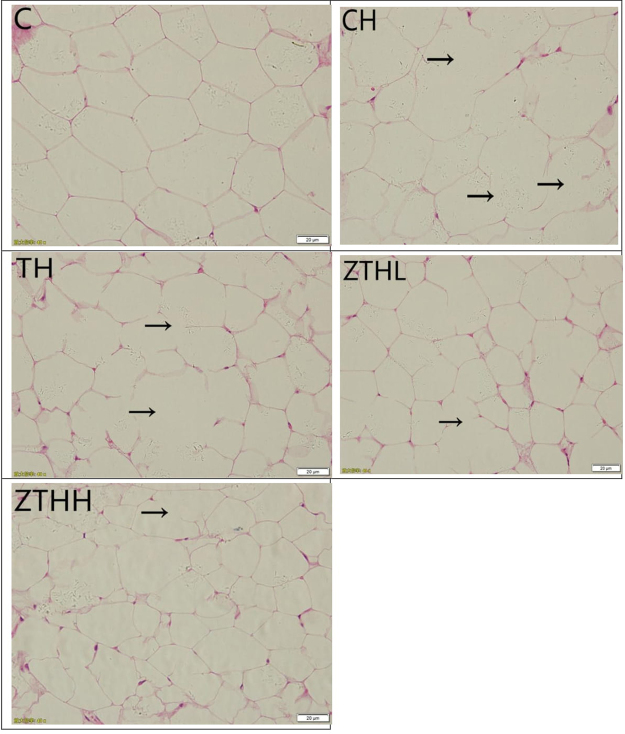
The histological features of adipose tissue in experimental rats (400×). Fat tissue of SD rats was stained with hematoxylin and eosin. Arrow: adipocyte incorporation.

**Figure 11:**
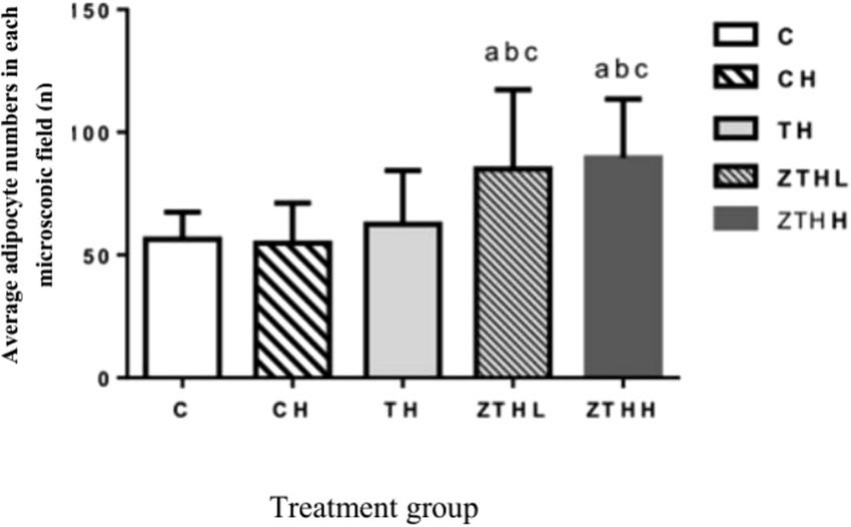
Comparison in the number of adipocyte in different treatment groups in histological examination. ^a^Vs C-group, *p* < 0.05; ^b^vs CH-group, *p* < 0.05; ^c^vs TH-group, *p* < 0.05.

In the C-group, the size of the adipocytes was normal, the cell nucleus was in the center, and the cytoplasm was uniform. These adipocytes were significantly increased in size in the CH-group TH-group, ZTHL-group, and ZTHH-group versus the C-group. There was increased size of adipocytes or hypertrophy in the CH-group versus the C-group. Adipocyte sizes were increased in the TH-group compared to both the ZTHL-group and the ZTHH-group. There were no significant morphological differences between the ZTHL-group and the ZTHH-group (Fig. 10). In addition, the numbers of adipocytes in both ZTHL and ZTHH groups were more than those in the control, CH and TH groups (p < 0.05) (Fig. 11).

## Discussion

We investigated the potential beneficial influence of Chinese Fenggang zinc selenium tea on glucose and lipid metabolism disorders and liver injury. The results of this study showed that Fenggang zinc selenium tea can combat glucose and lipid metabolic disorders and improve hepatic function. To the best of our knowledge, this was the first study to evaluate the beneficial effects of Chinese Fenggang zinc selenium tea on endocrine metabolism in high-sucrose-high-fat diet-induced obese rats.

### Effect of zinc and selenium tea on blood lipid and blood glucose

A diet high in sucrose and fat plays a major role in the onset and progression of metabolic disorders, including non-alcoholic fatty liver disease (NAFLD) and MetS^26^. Zhou *et al*. (2014) reported that high-fat diet-fed rats orally administered Jing-wei fu tea experienced a decrease in obesity-induced hepatic steatosis by regulating hepatic lipogenesis and lipolysis^27^. A study in adults in Poland demonstrated that high tea consumers (1 cup/day) had lower body mass indexes (BMIs) and waist circumferences. Tea consumption was negatively associated with MetS^28^. In addition, in a randomized controlled trial, Yuko *et al*. (2017) demonstrated that Japanese green tea may lower low-density lipoprotein cholesterol, and the magnitude of the lipid-lowering effect of the tea was significantly larger than that of placebo^29^. In this study, we designed a HSHF diet animal group via mimicking human diet habits. Alterations in the body weight of rats in the experimental groups revealed that tea can suppress the increases in the body weight of rats. The levels of TG and TC were lower in the zinc selenium tea group than in the normal green tea group. Morphological examination results indicated that the extent of pathologic changes in the liver and fat tissue were less extensive in the zinc selenium tea group versus the normal green tea group. In all, the antagonistic effect of zinc selenium tea on a HSHF diet was pronounced. The results were in accordance with previous lipometabolism studies^27–29^.

In the current study, a treatment dose of 0.24 g/kg/day (low-dose zinc selenium organic tea) amounts to 12 g of tea consumed per person per day, whereas the treatment dose of 1.20 g/kg/day (high-dose zinc selenium organic tea) amounts to the 60 g of tea consumed per person per day. The antagonistic effect on MetS, induced by the HSHFD in the ZTHL-group, was more prominent than in the ZTHH-group, suggesting a dose-response relationship between tea consumption and blood glucose and lipid levels, which may be associated with the enrichment of zinc and selenium in FengGang green tea. Zinc is involved in the structure, regulation, and activity of thousands of proteins and participates in many biological processes, such as redox regulation and signal transduction^30^. Selenium exerts its biological function through antioxidant enzymes, such as glutathione peroxidase (GPx)^31^. Zinc and selenium have been shown to possess antioxidant properties and have integral roles in numerous biological processes, including regulating blood lipid and glucose levels. From a biochemical measurement point of view, normal green tea and zinc selenium organic tea both improve effects on blood lipids. From a quantitative view, the blood lipid depressing effects of zinc selenium organic tea are better the effects of normal green tea.

### Effects of zinc and selenium tea on HI and bilirubin metabolism

The HI has long been used as a representative marker of hepatic injury^32^. Our results show that, compared with the CH-group, the HI was markedly decreased in the ZTHL-group (0.24 g/kg/day), while the HI of rats gavaged with low-dose zinc selenium tea did not increase. Our data supports the notion that zinc selenium tea has protective effects on liver function in experimental rats, the effects of which are stronger/better than normal green tea. From the point of view of AST activity, a significant reduction in serum AST levels was observed in the ZTHL-group versus the CH-group, which revealed the inhibitory effects of zinc selenium tea on the elevation of serum AST levels caused by a high-sucrose-high-fat diet, consistent with previous reports^33^. Li reported a protective effect of zinc threonine against liver injury in diabetic rats. Adequate intake of micronutrient zinc is thought to be beneficial for maintaining liver health. A selenium-enriched diet provides cellular protection against oxidative stress^31^. Therefore, zinc selenium tea has protective effects on HSHFD-induced liver damage in animals. The mechanism may be that zinc selenium tea can improve the oxidative stress status and restrain lipid peroxidation to protect liver from damage^7^.

TBIL, DBIL, and IBIL are three common biomarkers of bilirubin metabolism. Studies indicate that elevated serum TBIL, DBIL, and IBIL levels are associated with the fatty degeneration of liver cells, and disorders in bilirubin metabolism^33,34^. The serum levels of DBIL in the ZTHL-group were significantly higher compared to the CH-group in our study, which highlights the beneficial effects of low-dose zinc selenium tea on bilirubin metabolism in high-sucrose-high-fat diet-induced obese rats.

### Effects of zinc and selenium tea on cholestasis

Previous reports have suggested that TBA may be a more precise marker of cholestasis in chronic cholestatic liver disease^35,36^. Recent scientific literature has proposed that TBA can also function as an endocrine signaling molecule that activates multiple nuclear and membrane receptor-mediated signaling pathways, playing key roles in regulating TBA, glucose and lipid homeostasis, as well as energy balance^37^. A high fat, high sucrose diet can increase serum activities of transaminases and ALP and increase serum concentrations of TBA^38^. In the present study, an obvious depressive role of zinc selenium tea was observed in reducing the serum levels of TBA in high-sucrose-high-fat diet rats, which is in agreement with the findings of Sohair and Sary (2015).

A growing body of evidence shows that serum ALP is a marker of cholestasis^38–40^. Cholestasis develops from a defect in bile synthesis, impairment in bile secretion, or obstruction to bile flow, and is characterized by elevated serum ALP^39^. The data obtained from the present study indicates that zinc selenium tea improved ALP levels better than normal green tea. Zinc selenium tea at a dose of 0.24 g/kg/d can regulate bile acid metabolism by reducing the levels of ALP. The regulatory function of zinc selenium tea could be largely explained by its organic zinc and organic selenium content.

Our experimental results illustrated that the protective effect of zinc selenium tea against endocrine metabolism disorder which induced by high-sucrose-high-fat diet was better than that of green tea. Meanwhile several limitations of the present study need to be acknowledged. First, polyphenol in tea was not detected. Second, only blood glucose detection rather than the insulin level measurement in response to the OGTT was conducted. Third, we did not delve into the mechanism which was associated with the protective effects of Chinese Fenggang zinc selenium tea on metabolic syndrome. Despite these limitations, our study gives an overview of the beneficial effects of Chinese Fenggang zinc selenium tea on endocrine metabolism in high-sucrose-high-fat diet-induced obese rats.

## Conclusion

Zinc selenium tea can improve liver function in high-sucrose-high-fat diet rats. This result is in agreement with several studies that found zinc threonine can protect the liver from oxidative damage and improve the liver function of diabetic rats^33^. Selenium-containing green tea also has higher antioxidant and prebiotic activities than regular green tea^40^. Zinc is necessary for proper liver function since it has important antioxidant, anti-inflammatory, and anti-apoptotic properties^41^. Selenium may modulate a broad spectrum of key biological processes, including the cellular response to oxidative stress^42^. We conclude that zinc selenium tea showed a protective effect against hepatic damage. Therefore, zinc selenium tea can be used as a potential healthy drink for prevention and/or treatment of MetS and other diseases.

## References

[1] Hidayat S, Dhadhang WK, Lantip R (2017). Biochemical and histopathological effects of green tea nanoparticles in ironized mouse model. Res Pharm Sci..

[2] Alireza JM, Judith R, Catherine C, Marjan S, Tara Z (2015). The Role of Maternal Dietary Proteins in Development of Metabolic Syndrome in Offspring. Nutrients..

[3] Snoussi C (2014). Green tea decoction improves glucose tolerance and reduces weight gain of rats fed normal and high-fat diet. J Nutr Biochem..

[4] Higuchi N, Hira T, Yamada N, Hara H (2013). Oral administration of corn zein hydrolysate stimulates GLP-1 and GIP secretion and improves glucose tolerance in male normal rats and Goto-Kakizaki rats. Endocrinology..

[5] Kavita S, Neerja R, Khushboo G, Saurabh S (2017). Probable benefits of green tea with genetic implications. J Oral Maxillofac Pathol..

[6] Pan N, Liu YB, Zhang XY, Yu YH, Xiang XL (2015). The investigation of zinc and selenium content in Fenggang zinc and selenium tea. Science and Technology of West China..

[7] Shen X, Nie L, Liu H, Wu N (2014). Effects of zinc and selenium rich green tea of fenggang in guizhou province on mouse antioxidant ability. Journal of Guiyang Medical College..

[8] Liu H, Shen X, Nie L, Wu N (2014). Study on Influence of Se-enriched and Zinc-enriched Green Tea on Anti-oxidation and Anti-aging of Mice in Guizhou. Journal of Anhui Agri. Sci..

[9] Sigal S, Aliza HS, Zecharia M (2015). Nutrition Targeting by Food Timing: Time-Related Dietary Approaches to Combat Obesity and Metabolic Syndrome. Adv Nutr..

[10] Hae DW, Aesun S, Jeongseon K (2014). Dietary Patterns of Korean Adults and the Prevalence of Metabolic Syndrome: A Cross-Sectional Study. PLoS One..

[11] Gu D (2005). Prevalence of the metabolic syndrome and overweight among adults in China. Lancet..

[12] Juan AP (2016). Nutrition, insulin resistance and dysfunctional adipose tissue determine the different components of metabolic syndrome. World J Diabetes..

[13] Steven KM (2012). Exercise training with weight loss and either a high or low glycemic diet reduces metabolic syndrome severity in older adults. Ann Nutr Metab..

[14] Monika C, Helena D, Eliska P, Zuzana P, Ludmila K (2012). The Opposite Effects of High-Sucrose and High-Fat Diet on Fatty Acid Oxidation and Very Low Density Lipoprotein Secretion in Rat Model of Metabolic Syndrome. J Nutr Metab..

[15] Priyanga R, Shehani P, Priyadarshani G, Prasad K, Godwin RC (2015). Zinc and diabetes mellitus: understanding molecular mechanisms and clinical implications. Daru..

[16] Tang X, Shay NF (2001). Zinc has an insulin-like effect on glucose transport mediated by phosphorinositol-3-kinase and Akt in 3T3-L1 fibroblasts and adipocytes. J Nutr..

[17] Almugabgab, S. A. S. Relationship between plasma zinc levels and type 2 diabetes. Thesis for Master degree. Huazhong University of Science and Technology, Tongji Medical College, 2013.

[18] Roussel AM (2003). Antioxidant effects of zinc supplementation in Tunisians with type 2 diabetes mellitus. J. Am. Coll. Nutr..

[19] Jamilian M (2015). Metabolic response to selenium supplementation in women with polycystic ovary syndrome: a randomized, double-blind, placebo-controlled trial. Clin Endocrinol (Oxf)..

[20] Park K, Seo E (2016). Association between Toenail Mercury and Metabolic Syndrome Is Modified by Selenium. Nutrients..

[21] Arnaud J (2012). Gender differences in copper, zinc and selenium status in diabetic-free metabolic syndrome European population - the IMMIDIET study. Nutr Metab Cardiovasc Dis..

[22] Marco V (2014). Selenium for preventing cancer. Cochrane Database Syst Rev..

[23] Puchau B, Zulet MA, Gonzalez dEA, Navarro-Blasco I, Martinez JA (2009). Selenium intake reduces serum C3, an early marker of metabolic syndrome manifestations, in healthy young adults. Eur J Clin Nutr..

[24] Wu Shurong. The study of the relationships between serum elements and diabetes and dyslipidemia in the population of Pingyin. Thesis for Master degree. Shandong university, 2012.

[25] Li MZ (2016). Effects of zinc selenium tea on liver function in high fat and high sugar fed rats. Journal of Zunyi Medical University..

[26] Marventano S (2016). Coffee and tea consumption in relation with non-alcoholic fatty liver and metabolic syndrome: A systematic review and meta-analysis of observational studies. Clinical Nutrition..

[27] Jie Z, Liang Z, Jingsong Z, Xiaochun W (2014). Aqueous extract of post-fermented tea reverts the hepatic steatosis of hyperlipidemia rat by regulating the lipogenic genes expression and hepatic fatty acid composition. BMC Complement Altern Med..

[28] Giuseppe G (2015). Association of daily coffee and tea consumption and metabolic syndrome: results from the Polish arm of the HAPIEE study. Eur J Nutr..

[29] Yuko I (2017). Randomized controlled trial of the effects of consumption of ‘Yabukita’ or ‘Benifuuki’ encapsulated tea-powder on low-density lipoprotein cholesterol level and body weight. Food Nutr Res..

[30] Mariea DB (2010). Zinc and Zinc Transporter Regulation in Pancreatic Islets and the Potential Role of Zinc in Islet Transplantation. Rev Diabet Stud..

[31] Rayman MP (2000). The importance of selenium to human health. Lancet..

[32] Koul A, Ghara AR, Gangar SC (2006). Chemomodulatory effects of Azadirachta indica on the hepatic status of skin tumor bearing mice. Phytother Res..

[33] Li C (2011). Protective Effect of Zinc Threonine against Liver Injury in Diabetic Rats. Food Science..

[34] Gómez SA, Bexfield N, Scase TJ, Holmes MA, Watson P (2014). Total serum bilirubin as a negative prognostic factor in idiopathic canine chronic hepatitis. J Vet Diagn Invest..

[35] Ian A, Bouchier D, Pennington R (1978). Serum Bile Acids in Hepatobiliary Disease. Gut..

[36] Eva S, Milan J (2013). New insights in bilirubin metabolism and their clinical implications. World J Gastroenterol..

[37] Grasselli E (2014). Altered oxidative stress/antioxidant status in blood of alcoholic subjects is associated with alcoholic liver disease. Drug Alcohol Depend..

[38] Sohair MMR (2015). Effect of a high fat, high sucrose diet on the promotion of non-alcoholic fatty liver disease in male rats: the ameliorative role of three natural compounds. Lipids Health Dis..

[39] Arshad HR, Fahad MAS, Khaled SA, Salah MA, Masood AK (2015). Implications of Green Tea and Its Constituents in the Prevention of Cancer via the Modulation of Cell Signalling Pathway. Biomed Res Int..

[40] Poupon R (2015). Liver alkaline phosphatase: a missing link between choleresis and biliary inflammation. Hepatology..

[41] Molana. AL, Flanaganb J, Weic W, Moughanb PJ (2009). Selenium-containing green tea has higher antioxidant and prebiotic activities than regular green tea. Food Chemistry..

[42] Zaidi SNF, Mahboob T (2014). Protective role of Zinc in liver cirrhosis: study in rats. World J Pharm Sci..

